# Mechanisms of Anticancer Therapy Resistance: The Role of Cancer Stem Cells

**DOI:** 10.3390/ijms21239006

**Published:** 2020-11-27

**Authors:** Julhash U. Kazi

**Affiliations:** Division of Translational Cancer Research and Lund Stem Cell Center, Department of Laboratory Medicine, Lund University, 22336 Lund, Sweden; kazi.uddin@med.lu.se

Despite incredible progress in anticancer therapy development, resistance to therapy is the major factor limiting the cure of cancer patients. It results in the majority of cancer-related deaths in various types of cancer. Therefore, the identification of the factors that drive the development of therapy resistance is a pressing issue in the field. Cancer stem cells represent a small subpopulation of stem-like cells within the tumor that display characteristics of both cancer cells and stem cells and are resistant to chemotherapy and radiotherapy.

The stem cells in normal tissues are characterized by the capacity for self-renewal and the ability to differentiate into the specialized end cells of that tissue. Because stem cells are the longest-living cells in many tissues, the possibility exists that they can accumulate mutations over time. This idea is supported primarily by the clonal hematopoiesis, an expansion of blood cells derived from a mutated single hematopoietic stem cell, which is frequently observed in the elderly population. An example includes the frank acute myeloid leukemia blasts, in which normal hematopoietic stem cells carry one or two somatic mutations [1]. Furthermore, several studies from solid tumors suggest that the initial oncogenic mutations occur in stem cells [2,3]. However, a single oncogenic mutation is not enough to develop cancer. For example, the introduction of an oncogenic version of the Feline McDonough Sarcoma (FMS)-like tyrosine kinase 3 (FLT3), FLT3-ITD into mouse hematopoietic progenitor cells develops a myeloproliferative disease but not to leukemia [4]. FLT3 is a frequently mutated gene in acute myeloid leukemia (AML) and is also found to be mutated sporadically in other types of leukemia [5,6]. The subsequent mutations required for cancer cell transformation might happen in either the stem cell or the immature progenitor cell [7,8]. Several epigenetic regulators such as ten-eleven translocation 2 (TET2), DNA methyltransferase 3A (DNMT3A) and isocitrate dehydrogenase 1 (IDH1) and IDH2 are found to be mutated early on, which leads to activation of the self-renewal program but blocks differentiation [9]. Then, loss-of-function mutations in tumor suppressor genes (TP53, CDKN2A, etc.) lead to the inactivation of apoptosis and senescence or gain-of-function mutations in oncogenes (FLT3, KIT, RAS, etc.), resulting in an uncontrolled proliferation and expansion capacity [10].

Besides the self-renewal and differentiation capabilities, cancer stem cells exhibit tumor initiation capacity and long-term repopulation potential and thereby confer multidrug resistance, radiation resistance, tumor relapse and metastasis. Although the initial evidence regarding the cancer stem cells came from studies in leukemia [11], recent studies suggest the presence of cancer stem cells in several solid tumors including breast cancer, pancreatic cancer, prostate cancer, colorectal cancer, skin cancer and glioblastoma [12]. 

It is evident that cancer stem cells can originate from stem cells, progenitors and mature cells [13] (Figure 1A). The development of cancer stem cells requires multiple genetic and epigenetic changes and a favorable microenvironment. Somatic cells can be reprogrammed to pluripotent stem cells by transient ectopic overexpression of stem cell transcription factors such as Nanog, OCT4, KLF4, MYC and SOX2, which are also expressed by cancer stem cells to maintain stemness characteristics [14]. OCT4 is a homeodomain transcription factor reported to be highly expressed in cancer stem cells and its expression level is positively correlated with tumor grade, self-renewal capacity and tumorigenicity [15,16]. SOX2 plays an important role in the early development and maintenance of undifferentiated embryonic stem cells and is involved in the self-renewal and tumorigenesis of cancer stem cells while it inhibits differentiation. An elevated expression of Nanog is associated with tumor metastasis and predicts poor prognosis. KLF4 acts as a tumor suppressor or oncogene depending on the cancer type [14]. 

Signaling pathways that regulate self-renewal, differentiation, proliferation and survival in normal stem cells are similarly regulated in cancer stem cells [14]. For instance, the activation of the Wnt/β-catenin pathway maintains the quiescence state of stem cells and is important for the self-renewal of embryonic stem cells, and is also active in cancer stem cells [17]. The activation of Notch signaling promotes self-renewal and cell survival in cancer stem cells. Hedgehog signaling in cancer stem cells is important for maintenance, self-renewal, proliferation and tumorigenesis [18]. Several other pathways including NF-κB signaling, JAK-STAT pathway, TGFβ-SMAD signaling, PI3K pathway and PPAR signaling play important roles in cancer stem cell self-renewal, survival and proliferation [14,19]. Therefore, targeted inhibition of specific cancer stem cell signaling pathways can add value in cancer treatment.

Cancer stem cells display resistance to chemotherapy or radiotherapy-induced cell death [19,20,21,22,23,24] and, thus, can form new tumors (Figure 1B). Almost all chemotherapies target rapidly proliferating cells by inducing DNA damage or by inhibiting mitotic division [25]. During drug treatment, cancer stem cells can enter into a dormant state and thereby avoid chemotherapy-induced apoptosis [26]. Several factors including cell cycle regulatory genes, angiogenesis, hypoxia and tumor microenvironment play important roles in keeping the cells in this state [27]. Furthermore, cancer stem cells show deregulation of several cellular signaling pathways [28]. Activation of anti-apoptotic pathways such as PI3K, Wnt/β-catenin and Notch signaling can provide survival benefit [29]. Increased expression of ATP-binding cassette (ABC) transporters (ABCB1, ABCC1 and ABCG2) helps cancer stem cells to pump out drugs [30]. Cancer stem cells can undergo epithelial-to-mesenchymal transition (EMT) and EMT is associated with drug resistance [29]. Cancer stem cells actively interact with the microenvironment through paracrine factors, cell surface receptors and adhesion molecules. The cancer stem cell microenvironment plays an important role in maintaining its self-renewal capability and differentiation potential. Furthermore, the microenvironment protects cancer stem cells from chemotherapy-induced apoptosis and increases radiological tolerance [14]. Several agents targeting the cancer stem cell microenvironment have shown promising results in preclinical studies and clinical trials.

Current studies suggest that cancer stem cells contribute to tumorigenesis, metastasis, therapy resistance and relapse. Understanding the microenvironment and intracellular regulation of cancer stem cells is necessary for the identification of effective targeted therapies against cancer stem cells that might help to overcome anticancer therapy resistance.

## Figures and Tables

**Figure 1:**
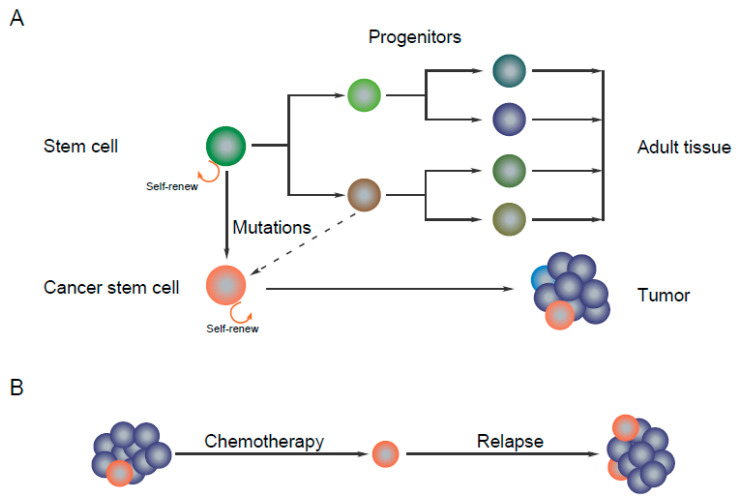
Cancer stem cells: (**A**) To form normal tissue, mature cells develop from the stem cell that differentiates to generate progenitors. Cancer stem cells develop from the normal stem cells or progenitors due to multiple mutations in oncogenes or tumor suppressors. Like normal stem cells, cancer stem cells also have self-renewal capacity. (**B**) Due to distinct drug-resistance properties, cancer stem cells can survive chemotherapy-induced cell death and thereby form new tumors.
